# Case report: Chronic inflammatory demyelinating polyradiculoneuropathy with severe central nervous system demyelination: a clinicopathological study

**DOI:** 10.3389/fimmu.2024.1477615

**Published:** 2024-12-05

**Authors:** Goichi Beck, Rika Yamashita, Makiko Kawai, Ryohei Yamamura, Tatsusada Okuno, Misa Matsui, Keiko Toyooka, Eiichi Morii, Hideki Mochizuki, Shigeo Murayama

**Affiliations:** ^1^ Department of Neurology, Osaka University Graduate School of Medicine, Suita, Japan; ^2^ Department of Pathology, Osaka University Graduate School of Medicine, Suita, Japan; ^3^ Department of Neurology, National Hospital Organization (NHO) Osaka Toneyama Medical Center, Toyonaka, Japan; ^4^ Brain Bank for Neurodevelopmental, Neurological and Psychiatric Disorders, Molecular Research Center for Children’s Mental Development, United Graduate School of Child Development, Osaka University, Suita, Japan; ^5^ Department of Neurology and Neuropathology (the Brain Bank for Aging Research), Tokyo Metropolitan Geriatric Hospital and Institute of Gerontology, Tokyo, Japan

**Keywords:** chronic inflammatory demyelinating polyradiculoneuropathy, spinal cord, white matter, demyelination, autopsy

## Abstract

Chronic inflammatory demyelinating polyradiculoneuropathy (CIDP) is an immune-mediated disease that mainly affects the peripheral nerves and nerve roots and typically presents with distal dominant motor and sensory disturbances as clinical symptoms. Central nervous system (CNS) demyelination with inflammation occurs infrequently in patients with CIDP. Here, we present a unique autopsy report of CIDP causing severe demyelination along the entire spinal cord. A Japanese woman exhibited progressive muscle weakness, muscle atrophy, sensory disturbances, and tremors in her upper and lower extremities, which began in her 60s. A nerve conduction study revealed a marked prolongation of distal latencies and very low or no compound muscle action potential amplitudes, and cerebrospinal fluid protein levels were prominently elevated. Following the diagnosis of CIDP, a combination of methylprednisolone pulse therapy, high-dose intravenous immunoglobulin therapy, and plasma exchange mildly improved her symptoms. The patient died of septic shock at the age of 74 years. Neuropathological examination revealed demyelinating lesions with inflammation in the peripheral regions of the anterior, lateral, and posterior funiculi along the entire spinal cord (from the cervical to the sacral cord), and axons and neurons were well preserved in these lesions. The nerve roots in the cervical and lumbar plexuses, cauda equina, sciatic nerve, and sural nerve showed prominent swelling and edema with infiltration of inflammatory cells. Many onion bulbs were visible in the fascicles of the sciatic and sural nerves. Our results suggest that demyelination with inflammation can occur in the CNS and peripheral nervous system in CIDP, especially in patients with specific conditions, such as severe intrathecal inflammation.

## 1 Introduction

Chronic inflammatory demyelinating polyradiculoneuropathy (CIDP) is a common neuropathy characterized by progressive and symmetrical muscle weakness and sensory disturbances in the distal parts of the upper and lower limbs (1–3). Phenotypic variants include distal acquired demyelinating symmetric (DADS) and multifocal acquired demyelinating sensory and motor (MADSAM) neuropathy (2). CIDP is usually diagnosed according to the European Federation of Neurological Societies/Peripheral Nerve Society guidelines, which are based on a combination of clinical features and electrophysiological criteria for peripheral nerve demyelination (3). On the other hand, previous neuroradiological studies have shown demyelination of the spinal cord, nerve roots, and peripheral nerves in patients with CIDP (4–6).

In the present study, we report an autopsy case of CIDP with severe demyelination along with inflammation of the peripheral regions of the entire circumference of the whole spinal cord. Severe intrathecal inflammation could cause demyelination of the spinal cord as well as of the peripheral nerves in CIDP.

## 2 Case presentation

### 2.1 Clinical summary

A Japanese woman in her 60s began to exhibit tremors in her bilateral upper extremities. She had a history of hypertension, cervical spondylosis, and paroxysmal atrial fibrillation. She had no history of alcohol intake, diabetes mellitus, and cancer chemotherapy. At the age of 68 years, the patient began to experience numbness in both upper extremities and gait disturbances. Subsequently, she began to exhibit sensory disturbances, including numbness and dysesthesia in the distal part of her lower extremities and visited the neurology department at the age of 71. The patient showed no fever of unknown origin, and no mucocutaneous manifestations and swollen lymph nodes were found. Neurological examinations revealed muscle weakness and atrophy, dysesthesia, hyperalgesia, marked reduction in vibration and position sense in the upper and lower extremities, and loss of the deep tendon reflex (**Supplementary Table 1**). The patient showed postural and kinetic tremor in her bilateral hands and diagnosed with essential tremor temporarily, however, drug therapy such as propranolol and clonazepam had no effect. She showed no micturition, defecation, or sexual dysfunction. A nerve conduction study (NCS) revealed marked prolongation of distal latencies (DLs) and very low compound muscle action potential (CMAP) amplitudes in the right median and radial nerves. No CMAPs were evoked in the tibial nerve. Sensory nerve action potentials (SNAP) were not evoked in the right median or sural nerves. Cerebrospinal fluid (CSF) tests revealed markedly elevated protein levels (490 mg/dL, normal range <40). The blood test results, including complete blood count, serum biochemistry, autoantibodies, vitamin levels and tumor markers, were normal (**Supplementary Table 2**). M protein was not detected in the immunofixation electrophoresis. Abdominal skin punch biopsy detected no evidence of amyloidosis and lymphoma. Sural nerve biopsy revealed prominent edematous changes in the subperineurial space of 5 out 9 nerve fascicules, and edematous changes were relatively mild in the other four nerve fascicles. A reduced number of myelinated fibers and formation of onion bulbs were also visible in the nerve fascicles (**Supplementary Figure 1**). Genetic tests revealed no pathogenic mutations in hereditary neuropathy-associated genes, including PMP22 (**Supplementary Table 3**). Finally, the patient was diagnosed with CIDP and received methylprednisolone pulse therapy and high-dose intravenous immunoglobulin (IVIG) therapy. Although her clinical symptoms did not improve, the NCS showed improvement in the DLs, amplitudes of CMAPs, and conduction velocities (CVs) in her median and radial nerves. Two months later, the patient was transferred to the hospital for immunotherapy. She exhibited muscle weakness and atrophy. She also showed dysesthesia, hyperalgesia, marked reduction in vibration and position sense in the distal parts of his upper and lower extremities and extended to both elbows and knees, and a positive Romberg sign (**Supplementary Table 1**). She exhibited postural and kinetic tremors in her bilateral upper extremities, however, no parkinsonian symptoms including akinesia and rigidity were found. The patient showed no sign of cranial nerve palsy. CSF tests showed a normal cell count (4/mm^3^) but a marked elevation of in protein levels (625 mg/dL). In the NCS, sensory and motor responses were absent in the median, ulnar, tibial, and sural nerves. Tests for autoantibodies, including anti-ganglioside, anti-MAG (myelin-associated glycoprotein), anti-SGPG (sulfated glucuronyl paragloboside), anti-neurofascin155, and anti-contactin-1 antibodies, were all negative. Lumbar magnetic resonance imaging revealed prominent bilateral hypertrophy of the lumbar roots (**Supplementary Figure 2**). After admission, three courses of methylprednisolone pulse and seven plasma exchange treatments were administered; consequently, her motor symptoms improved, and CSF protein levels decreased (**Supplementary Table 1**). Subsequently, oral administration of prednisolone and cyclophosphamide was initiated. At the ages of 72 and 73 years, she was admitted to the hospital and received methylprednisolone pulse, high-dose IVIG, and plasma exchange therapies. Although the CSF protein levels decreased in response to immunotherapy, her motor and sensory symptoms did not improve (**Supplementary Table 1**). According to the results of sural nerve biopsy at age of 71, the patient could be already at an advanced stage before plasma exchange was started. At the age of 74 years, an initiation of rituximab treatment had been planned for advanced immunomodulatory therapy, however, a few weeks later she was admitted to the hospital as an emergency patient due to disturbance of consciousness and respiratory distress. Although antibiotic therapy was initiated for pneumonia, the patient died of septic shock after a total clinical course of approximately 10 years. An autopsy was performed 20 h and 58 min after death.

### 2.2 Pathological findings

The right hemisphere of the brain, some segments of the spinal cord, and right sciatic nerve were frozen for molecular and biochemical studies. The left hemisphere of the brain, rest of the spinal cord including nerve roots, and left sciatic nerve were fixed in 10% buffered formalin. The left sural nerve was fixed in 2.5% glutaraldehyde and embedded in epon, and 1-μm–thick toluidine blue-stained sections were prepared. Ultrathin sections were prepared, stained with uranyl acetate and lead citrate, and examined under a transmission electron microscope (H-7650; Hitachi High-Technologies Co., Tokyo, Japan). The patient’s left biceps brachii and tibialis anterior muscles were collected at autopsy and snap-frozen in acetone pre-cooled on dry ice to minimize cryoartifacts.

After macroscopic observation, appropriate areas were dissected and embedded in paraffin. Paraffin-embedded 6-μm–thick sections of the brain and spinal cord were stained with hematoxylin and eosin (HE) and Klüver-Barrera (KB) staining. Paraffin-embedded sections of the nerve roots, sciatic nerve, and sural nerve were stained with azan. For immunohistochemistry, the primary antibodies used were mouse monoclonal antibodies against CD68 (1:1000, clone PG-M1, Dako, Glostrup, Denmark) and phosphorylated neurofilaments (pNF; SMI31, Covance, Berkeley, CA, USA, 1:10000).

**Figure 1:**
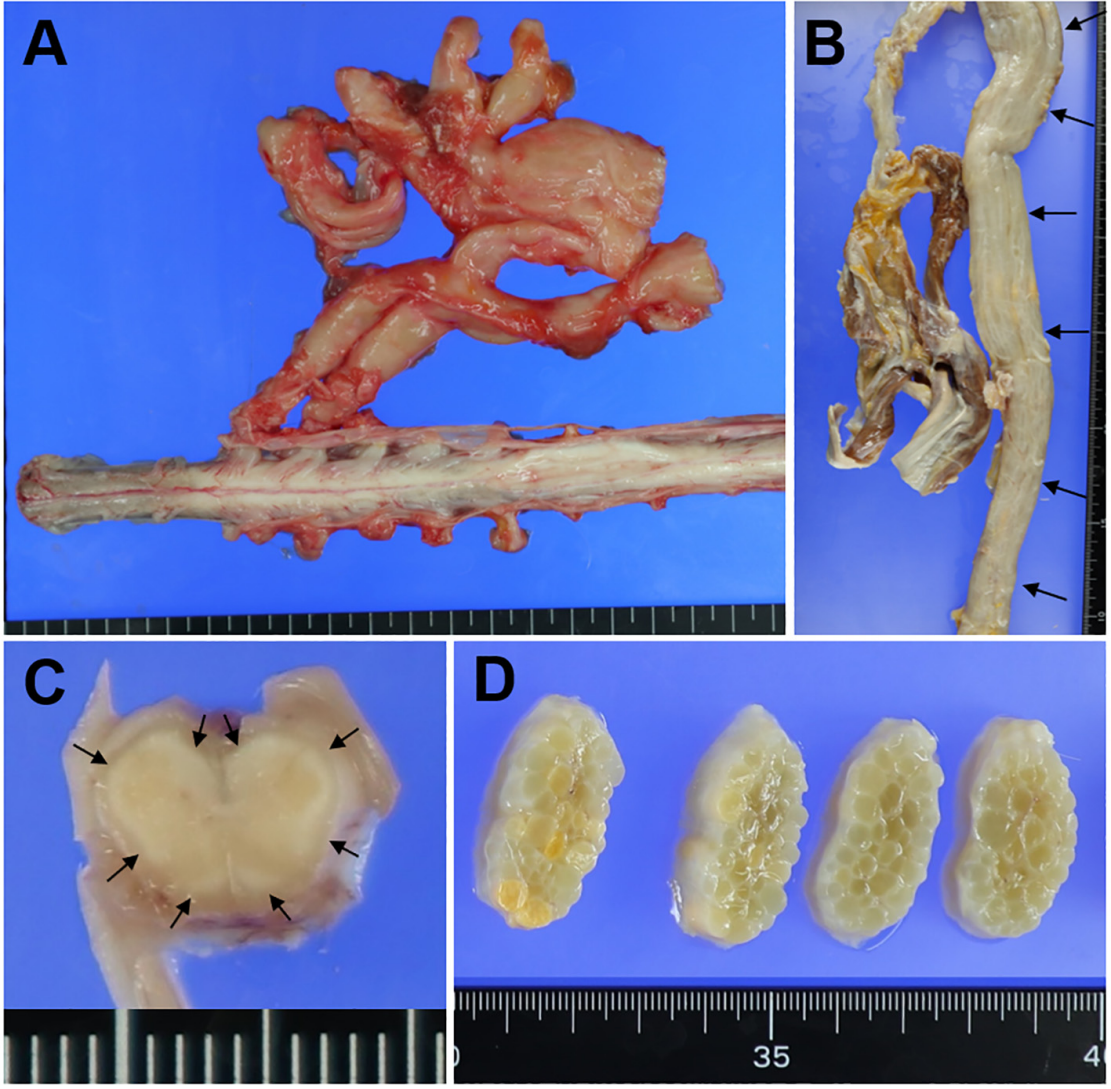
Macroscopic findings. Macroscopic findings in the lumbar plexus **(A)**, sciatic nerve **(B, D)**, and lumbar cord **(C)**. **(A)** The nerves in the lumbar plexus are prominently swollen. **(B)** The sciatic nerve is markedly swollen along its entire length (arrows). **(C)** In the lumbar cord section, the outer layers of the anterior, lateral, and posterior funiculi are whitish (small arrows). **(D)** In cross-sections of the sciatic nerve, prominent swelling of the nerve fascicles is visible.

The brain weighed 1200 g before fixation, and macroscopic observation revealed no obvious abnormal findings. By contrast, prominent swelling and hypertrophy of the nerves of the cervical and lumbar plexuses (**Figure 1A**), nerve roots, cauda equina, and peripheral nerves, including the sciatic nerves (**Figure 1B**), were visible. In the spinal cord sections, the outer layers were whitish (**Figure 1C**). In cross-sections of the sciatic nerves, prominent swelling of the nerve fascicles was visible (**Figure 1D**).

**Figure 2:**
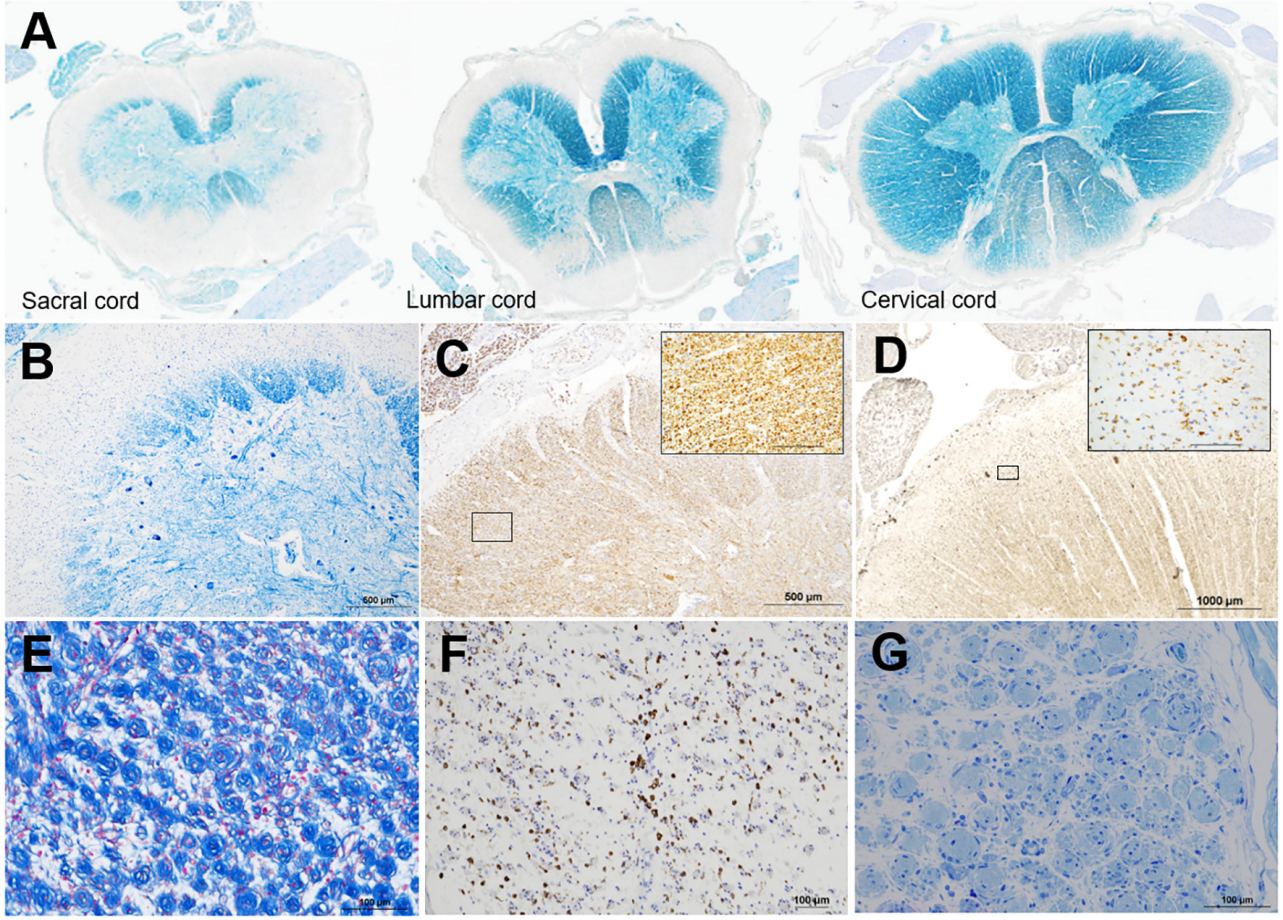
Semi-macroscopic and microscopic findings. Semi-macroscopic findings in the cervical, lumbar, and sacral cords **(A, B)**. In each section, myelin pallor is visible in the peripheral regions of the anterior, lateral, and posterior funiculi via KB staining **(A, B)**. Peripheral demyelination is most prominent in the caudal part of the spinal cord **(A)**. Microscopic findings in the sacral cord **(B-D)**, nerves in the lumbar plexus **(E, F)**, and sural nerve **(G)** are shown. In the sacral cord, where myelin pallor is most prominently visible in the peripheral areas, the anterior horn cells **(B)** and SMI31-positive axons **(C)** are well preserved. CD68-positive cells are infiltrated in the outset layer of the white matter **(D)**. The inlets are the high-magnification squares in **(C)** and **(D)**. In the lumbar plexus, many onion bulbs are visible **(E)**, and CD68-positive cells are prominently infiltrated **(F)**. A semi-thin section of the sural nerve shows edematous changes in the endoneurium, loss of large and small myelinated fibers, and formation of many onion bulbs **(G)**. KB staining **(A, B)**, azan staining **(E)**, and immunohistochemical staining for SMI31 **(C)** and CD68 **(D, F)**; toluidine blue staining **(G)**. The scale bars = 1000 μm **(D)**, 500 μm **(B, C)**, 100 μm (**E-G** and inlets in **C, D**).

KB staining revealed myelin pallor of the peripheral regions of the anterior, lateral, and posterior funiculi of the entire spinal cord, including the cervical, thoracic, lumbar, and sacral cords (**Figures 2A, B**). Peripheral demyelination of the sacral and lumbar cords was most prominent and that of the cervical cord was relatively milder (**Figure 2A**). By contrast, the anterior horn cells and SMI31-positive axons were preserved in severely demyelinating lesions in the spinal cord (**Figures 2B, C**). CD68-positive cells infiltrated the demyelinating lesions but were seen less frequently in the outset layer of the white matter (**Figure 2D**). Swelling of the nerve fascicles was visible in the cervical and lumbar plexuses. (**Figure 2E**), and a large number of onion bulbs were observed at higher magnification (**Figure 2F**). CD68-positive cells markedly infiltrated the nerve plexuses (**Figure 2G**). In both the sciatic and sural nerves, edematous changes were visible in the nerve fascicles, and normally myelinated fibers were completely lost. Numerous onion bulbs were observed in each fascicle (**Figure 2H**). Ultra-microscopic images revealed onion bulbs with more than 20 layers, bands of Bungner, and a marked loss of unmyelinated fibers (**Supplementary Figures 3A, B**). Large fiber-type grouping after denervation was visible in the biceps brachii muscle, and all fibers in the tibialis anterior muscle were extremely atrophic (**Supplementary Figures 4A, B**). Demyelination of the cranial nerves, including the trigeminal, facial, and glossopharyngeal nerves, was also found (**Supplementary Figures 5A–C**), while no demyelinating lesions were visible in the brain upon KB staining.

## 3 Discussion

Here, we report the clinical and neuropathological findings in a unique case of CIDP. Our case involved severe demyelination and infiltration of inflammatory cells in the peripheral regions of the circumference of the entire spinal cord, from the cervical to the sacral cord, as well as peripheral nerves and nerve roots. Extensive inflammation can spread to the white matter of the spinal cord in patients with CIDP.

In CIDP, demyelination caused by inflammation occurs mainly in peripheral nerves. The diagnosis of CIDP is heavily based on the presence of PNS demyelinating lesions accompanied by inflammation on electrophysiological and neuropathological examinations (7, 8). On the other hand, previous neuroradiological studies have shown demyelination of the CNS in some patients with CIDP (4–6, 9), and demyelinating lesions in the posterior funiculus have been reported in an autopsy case of CIDP (10). Although rare, previous reports have described compression myelopathy induced by nerve root hypertrophy in patients with CIDP (11, 12). Involvement of the spinal cord should be considered in the management of CIDP.

In the present case, demyelinating lesions with inflammation were visible in the peripheral areas of the anterior, lateral, and posterior funiculi and were found predominantly in the caudal parts of the spinal cord. Since axons and neuronal cells are well preserved in severely demyelinating lesions, CNS myelin could also be a target of inflammation in CIDP. In the present case, the CSF protein levels were extremely high (>900 mg/dL), and prominent swelling/edema with inflammation was visible in the nerve roots and peripheral nerves, suggesting the presence of chronic and severe inflammation of the intrathecal space; the white matter could be affected in patients with such conditions.

The involvement of the white matter of the spinal cord, such as spongy degeneration and vacuolar demyelination in peripheral areas, has been reported in patients with certain conditions, including CNS lupus (13), Burkitt’s lymphoma (14), and chemotherapy-induced myelopathy (15, 16). There may be a common factor that attacks the white matter of the spinal cord from the external side. Further pathological investigations of similar cases are required to elucidate the underlying mechanisms.

## Data Availability

The original contributions presented in the study are included in the article/**Supplementary Material**. Further inquiries can be directed to the corresponding authors.
