# Supplementary material for: Case report: Chronic inflammatory demyelinating polyradiculoneuropathy with severe central nervous system demyelination: a clinicopathological study
Source: Front Immunol. 2024 Dec 5;15:1477615. doi: 10.3389/fimmu.2024.1477615 (PMC11655328; doi:10.3389/fimmu.2024.1477615)
Supplement: Supplementary file 1 [file DataSheet1.docx]

Supplementary Material

**Chronic inflammatory demyelinating polyradiculoneuropathy with severe CNS demyelination: a clinicopathological study**

Goichi Beck, Rika Yamashita, Makiko Kawai, Ryohei Yamamura, Tatsusada Okuno, Misa Matsui, Keiko Toyooka, Eiichi Morii, Hideki Mochizuki, Shigeo Murayama

***Correspondence**:

Goichi Beck, M.D., Ph.D.

[g-beck@neurol.med.osaka-u.ac.jp](mailto:g-beck@neurol.med.osaka-u.ac.jp)

Shigeo Murayama, M.D., Ph.D.

[smurayam@bbarjp.net](mailto:smurayam@bbarjp.net)

This file includes:

Supplementary Figures 1-5

Supplementary Table 1-3

**Supplementary Figure 1.**

**Pathological findings of the sural nerve biopsy.**


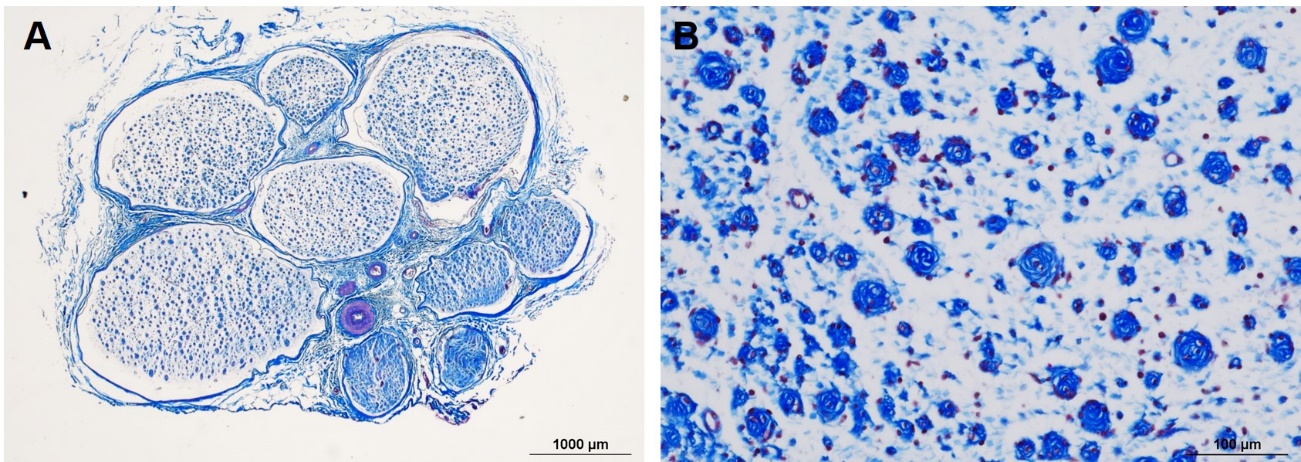


Azan staining in the sural nerve biopsy performed at age 71 shows edematous changes in the subperineurial space and endoneurium of 5 out of 9 nerve fascicles (A). With a higher magnification, normally myelinated fibers are lost and a lot of onion bulbs are visible (B). Scale bars = 1000 µm (A), 100 µm (B).

**Supplementary Figure 2.**

**Magnetic resonance images of the lumbar cord.**


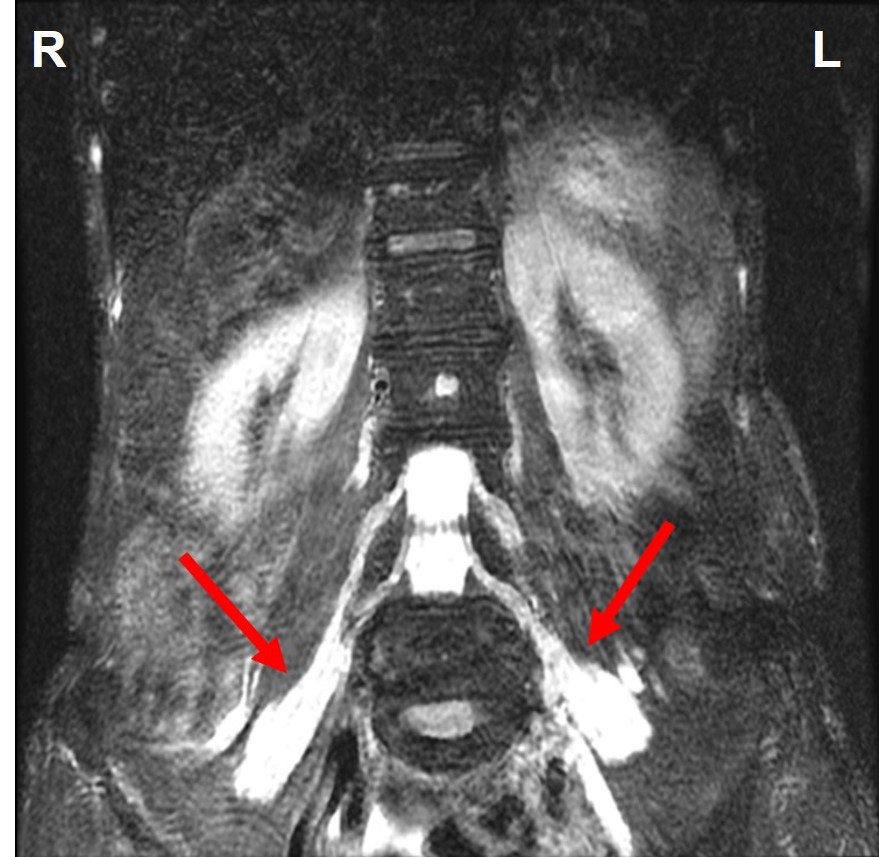


T2-weighted fat sat image of the lumbar cord shows swelling of the bilateral nerve roots (red arrows).

**Supplementary Figure 3.**

**Ultra-microscopic images the sural nerve.**


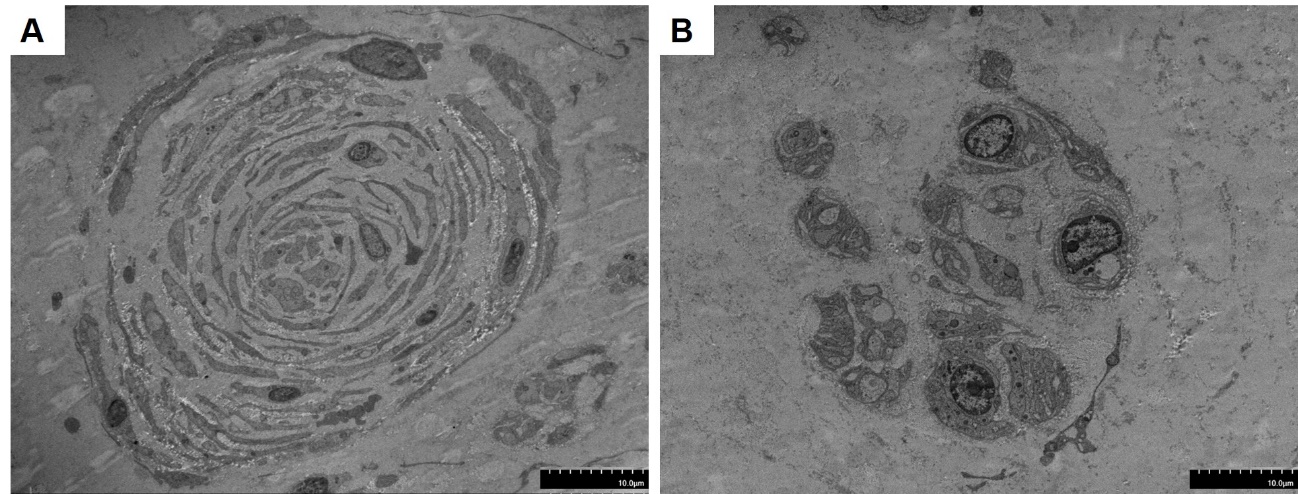


Ultra-microscopic image shows an onion bulb with more than 20 layers and Bungner bands (A). (B) Loss of unmyelinated fibers is visible. Scale bars = 10 µm.

**Supplementary Figure 4.**

**Microscopic findings in the biceps brachii and tibialis anterior muscles.**


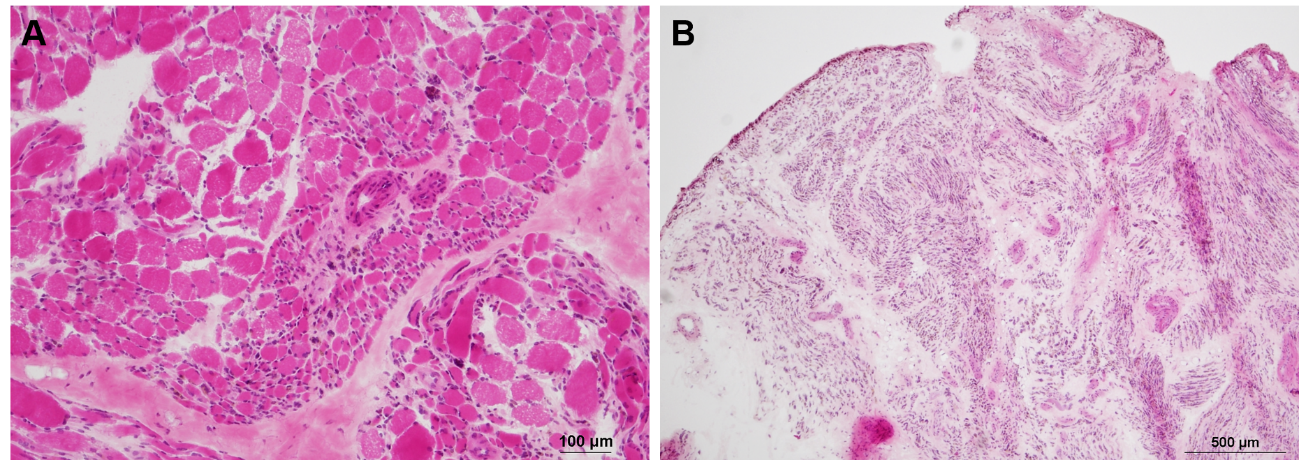


HE staining in the biceps brachii muscle (A) and tibialis anterior muscle (B) is shown. (A) Large fiber-type grouping after denervation is visible. (B) All fibers are extremely atrophic showing advanced neurogenic change. Scale bars = 100 µm (A), 500 µm (B).

**Supplementary Figure 5.**

**Microscopic findings in the trigeminal, facial and glossopharyngeal nerves.**


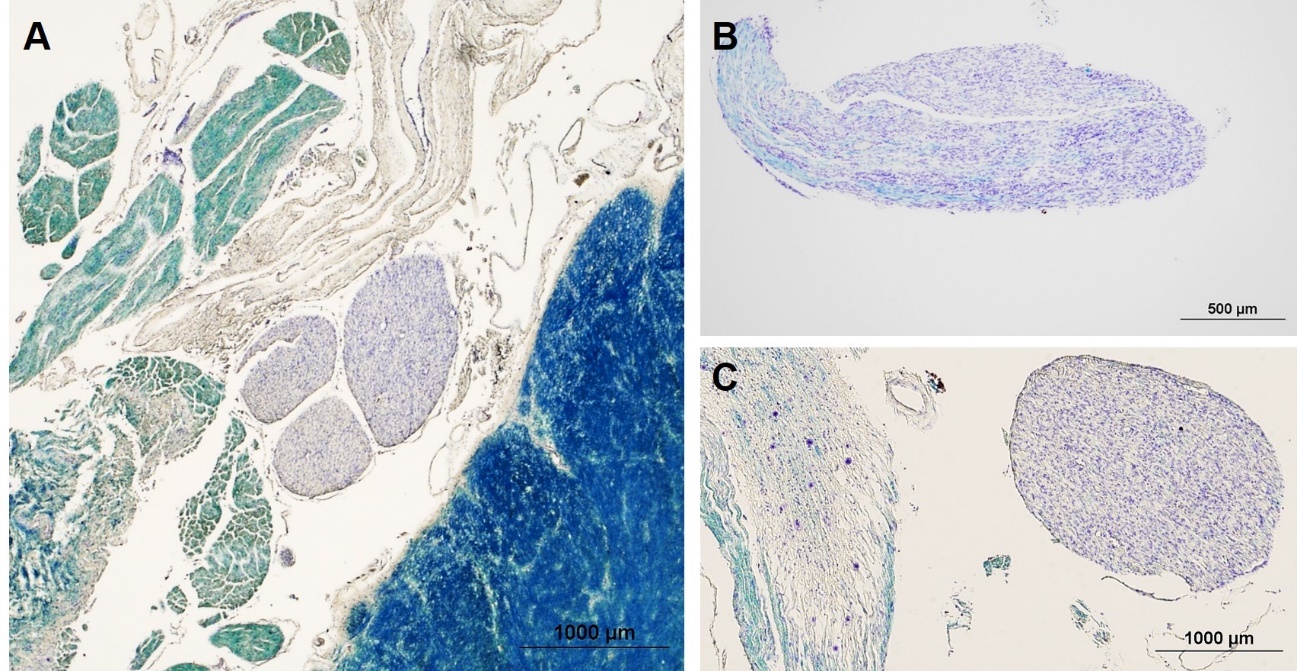


On KB staining, myelin pallor is visible in the trigeminal nerve (**A**), facial nerve (**B**) and glossopharyngeal nerve (**C**). Scale bars = 1000 µm (**A**, **C**), 500 µm (**B**).

**Supplementary Table 1.**

**Summary of the clinical course.**

|  | At age of 71  (initial admission) |  | After immunotherapy (#1) | At age of 73  (3^rd^ admission) |  | After immunotherapy (#2) |
| --- | --- | --- | --- | --- | --- | --- |
| MMT Deltoid  　　 Biceps  　　 Triceps  　　 Wrist extensor  　　 Wrist flexor  　　 APB  　　 FDP  　　 Iliopsoas  　　 Quadriceps femoris  　　 Hamstrings  　　 Tibialis anterior  CSF proteins (mg/dl) | 5/5  4/4  4/4  5/5  4/4  2/2  3/2  4/4  4/4  4/4  3/2  625 |  | 5/5  5/5  5/5  5/5  5/5  2/2  4/5  4/4  5/5  5/5  3/2  389 | 4-/4-  3/3  4-/4-  3/2+  3/3  0/0  3/3  4/4  4/4  3/3  2-/2-  712 |  | 4-/4-  3/3  4-/4-  3/2+  3/3  0/0  3/3  4/4  4/4  3/3  2-/2-  Not examined |

APB, abductor pollicis brevis; FDP, flexor digitorum profundus.

Immunotherapy #1 includes methylprednisolone pulse and plasma exchanges.

Immunotherapy #2 contains methylprednisolone pulse, high-dose IVIG, and plasma exchange.

**Supplementary Table 2.**

**Laboratory data at age of 71**

| Vitamin B1  Vitamin B12  Folic acid  TSH  Free T4  BNP  sIL-2R  Autoantibodies  ANA  Anti-RNP  Anti-SS-A  Anti-SS-B  Anti-Scl-70  C-ANCA  P-ANCA  anti-MAG  anti-SGPG  anti-neurofascin155  anti-contactin-1 | 4.2 µg/dL (2.6-5.8)  354 pg/mL (180-914)  5.3 ng/mL (4<)  0.61 µIC/mL (0.45-3.72)  1.6 ng/dL (0.8-1.7)  10.4 pg/mL (<40)  255 U/mL (121-613)  negative  negative  negative  negative  negative  negative  negative  negative  negative  negative  negative | Anti-ganglioside antibodies  GM1-IgM, IgG  GM2-IgM, IgG  GM3-IgM, IgG  GD1a-IgM, IgG  GD1b-IgM, IgG  GD3-IgM, IgG  GT1b-IgM, IgG  GQ1b-IgM, IgG  Gal-C-IgM, IgG  Tumor markers  CA19-9  NSE  CEA  SCC  AFP  CYFRA | negative  negative  negative  negative  negative  negative  negative  negative  negative  12.8 U/mL (0-35.4)  10.7 ng/mL (0-13.8)  3 ng/mL (0-5)  0.7 ng/mL (0-2)  3 ng/mL (0-7)  1.8 ng/mL (<2.1) |
| --- | --- | --- | --- |

Normal ranges in our institute are indicated in ( ).

TSH, thyroid-stimulating hormone; BNP, B-type natriuretic peptide; sIL-2R, soluble interleukin-2 receptor; ANA, antinuclear antibody; RNP, ribonucleoprotein; SS, Sjögren syndrome; ANCA, anti-neutrophil cytoplasmic antibody; CA, carbohydrate antigen; NSE, CEA, carcinoembryonic antigen; NSE, neuron-specific enolase; SCC, squamous cell carcinoma; AFP, alpha fetoprotein; CYFRA, cytokeratin 19 fragment

**Supplementary Table 3.**

**The list of genes examined in the present case.**

| *PMP22, AARS, APTX, ARHGEF10, DHH, DNM2, EGR2, FGD4, GAN, GARS, GDAP1, GJB1, HARS, HK1, HOXD10, HSPB1, HSPB8, KARS, LITAF, LMNA, MARS, MED25, MFN2, MPZ, MTMR2, NDRG1, NEFL, PMP22, PRPS1, PRX, RAB7A, SBF2, SETX, SH3TC2, SLC12A6, SOX10, TDP1, TRPV4, TTR, YARS, BSCL2, DCTN1, DHTKD1, DYNC1H1, FBLN5, FBXO38, GJB3, GNB4, HSPB3, IGHMBP2, INF2, KIFIA, LRSAM1, PDK3, REEP1, SBF1, SLC5A7, TFG, TRIM2, DCAF8, SURF1, SACS, GALC, PLEKHG5* |
| --- |
